# Innovations in Pediatric Therapeutics Development: Principles for the Use of Bridging Biomarkers in Pediatric Extrapolation

**DOI:** 10.1007/s43441-022-00445-6

**Published:** 2022-09-03

**Authors:** Thomas R. Fleming, Christine E. Garnett, Laurie S. Conklin, Solange Corriol-Rohou, Sudharshan Hariharan, Daphne Hsu, Guenther Mueller-Velten, Yeruk Mulugeta, Ronald Portman, Mark D. Rothmann, Norman L. Stockbridge, Simon Wandel, Jialu Zhang, Lynne Yao

**Affiliations:** 1 Department of Biostatistics, University of Washington, Seattle, WA USA; 2 Division of Cardiology and Nephrology, Office of New Drugs, Center for Drug Evaluation and Research, U.S. Food and Drug Administration, Silver Spring, MD USA; 3 Janssen Research and Development, Spring House, PA USA; 4 AstraZeneca, Paris, France; 5 Office of Clinical Pharmacology, Office of Translational Science, Center for Drug Evaluation and Research, U.S. Food and Drug Administration, Silver Spring, MD US; 6 Pediatric Heart Center, Children’s Hospital at Montefiore and Albert Einstein College of Medicine, Bronx, NY USA; 7 Novartis Pharma AG, Basel, Switzerland; 8 Division of Pediatrics and Maternal Health, Office of New Drugs, Center for Drug Evaluation and Research, U.S. Food and Drug Administration, Silver Spring, MD USA; 9 Novartis Pharmaceuticals, East Hartford, CA US; 10 Office of Biostatistics, Office of Translational Science, Center for Drug Evaluation and Research, U.S. Food and Drug Administration, Silver Spring, MD USA

**Keywords:** Biomarkers, Heart failure, Pediatric extrapolation, Pulmonary arterial hypertension

## Abstract

Even with recent substantive improvements in health care in pediatric populations, considerable need remains for additional safe and effective interventions for the prevention and treatment of diseases in children. The approval of prescription drugs and biological products for use in pediatric settings, as in adults, requires demonstration of substantial evidence of effectiveness and favorable benefit-to-risk. For diseases primarily affecting children, such evidence predominantly would be obtained in the pediatric setting. However, for conditions affecting both adults and children, pediatric extrapolation uses scientific evidence in adults to enable more efficiently obtaining a reliable evaluation of an intervention’s effects in pediatric populations. Bridging biomarkers potentially have an integral role in pediatric extrapolation. In a setting where an intervention reliably has been established to be safe and effective in adults, and where there is substantive evidence that disease processes in pediatric and adult settings are biologically similar, a ‘bridging biomarker’ should satisfy three additional criteria: effects on the bridging biomarker should capture effects on the principal causal pathway through which the disease process meaningfully influences ‘feels, functions, survives’ measures; secondly, the experimental intervention should not have important unintended effects on ‘feels, functions, survives’ measures not captured by the bridging biomarker; and thirdly, in statistical analyses in adults, the intervention’s net effect on ‘feels, functions, survives’ measures should be consistent with what would be predicted by its level of effect on the bridging biomarker. A validated bridging biomarker has considerable potential utility, since an intervention’s efficacy could be extrapolated from adult to pediatric populations if evidence in children establishes the intervention not only to be safe but also to have substantive effects on that bridging biomarker. Proper use of bridging biomarkers could increase availability of reliably evaluated therapies approved for use in pediatric settings, enabling children and their caregivers to make informed choices about health care.

## Introduction

Prior to the late 1990s, most drugs and biological products used to treat children were not FDA-approved for use in pediatric patients (1). Over the last two decades, the availability of approved therapies to treat conditions in children has increased greatly. Over 900 prescription drug and over-the-counter products now contain pediatric-specific labeling information. Much of this success is due to the passage of important legislation in the United States that promotes and requires pediatric studies (2, 3). Importantly, approval of prescription drug and biological products for use in children follows the same high standards as that in adults, requiring demonstration of substantial evidence of effectiveness and a favorable benefit–risk profile (4). Special ethical requirements must be followed for inclusion of children in clinical research, including that children should not be enrolled in clinical research unless their participation is required to obtain the necessary information (5). In diseases and conditions that primarily affect children, (e.g., rare pediatric cancers and diseases of the neonate), enrollment of sufficient numbers of pediatric patients is needed to obtain substantial evidence of effectiveness and adequate safety information because the disease does not occur in adults. However, for conditions that affect both adults and children, it is most often appropriate to initiate the intervention in adults first. In this situation, pediatric extrapolation, when scientifically valid, may be used to reduce the level of dependence on a pediatric clinical study. Pediatric extrapolation has been defined as “*an approach to providing evidence in support of effective and safe use of drugs in the pediatric population when it can be assumed that the course of the disease and the expected response to a medicinal product would be sufficiently similar in the pediatric and reference (adult or other pediatric) population”* (5). Thus, once a drug or biological product has been approved in adults, efficacy data could be leveraged to support approval in a pediatric population if there is evidence that the disease and response to intervention are similar between adult and pediatric populations. The choice of pediatric trial design will depend on the quality of data that supports similarity of disease and response to intervention between the adult (reference) and pediatric (target) populations. In most cases, additional safety data will be needed from the target pediatric population to support a benefit–risk assessment.

The enhanced understanding and experience in conducting and analyzing pediatric clinical trials as well as improved use of statistical and pharmacological modeling approaches to analyze data over the last 20 years has advanced the use of pediatric extrapolation. The appropriate use of pediatric extrapolation and the type and amount of data to be generated must be carefully considered and agreed upon with regulatory authorities.

The goal of the 2021 Advancing the Development of Pediatric Therapeutics (ADEPT) 7 workshop, Advancing Complex Innovative Trial Designs to Accelerate Drug Development in Pediatric Patients, was to review and discuss the role of bridging biomarkers and innovative trial designs that can be used as part of a pediatric extrapolation approach (6). Bridging biomarkers can make full use of available existing data to minimize the need to generate new data, avoid unnecessary studies in children, and ultimately improve the efficiency of pediatric therapeutics development. The focus of this paper is to describe the use of biomarkers, with emphasis on the unique role of bridging biomarkers for pediatric extrapolation.

## Pediatric Extrapolation

Pediatric extrapolation relies on an evaluation of similarity in disease course and response to interventions between adult and pediatric populations such that adult data can be used to inform drug development in pediatrics. Approaches to pediatric extrapolation have been described in various regulatory guidance documents (5, 7, 8) and vary based on the level of confidence in the evidence to support similarity. For example, matching an established effective exposure in adults to identify an effective dose in children can be considered when there is high confidence that the course of disease and response to intervention are similar between adult and pediatric populations. When the course of disease is similar, but the response to invention in pediatrics is not known or differs from adults, dose–response studies of clinical endpoints or bridging biomarkers can be considered. When there is low confidence in the similarity of disease and response to an intervention, adequate and well-controlled study(ies) in children will generally be needed.

### Some Uses of Biomarkers in Pediatric Extrapolation

As in overall drug development, response biomarkers are used in pediatric drug development (Table 1). Pharmacodynamic (PD) biomarkers can be used to support dose selection for later phase trials, particularly in conditions with smaller populations (e.g., rare diseases). In a first-in-pediatrics dose ranging study of vamorolone in 48 boys with Duchenne muscular dystrophy, seven serum biomarkers reflective of inflammation were selected. The biomarkers were anticipated to reflect the drug’s mechanism of action, as supported by preliminary studies. Six of the seven biomarkers were dose responsive in the study and were considered when selecting doses for future trials (9, 10). In a dose ranging study of dalteparin in children with venous thromboembolism, doses were adjusted to achieve the target anti-Xa level. A population pharmacokinetic (PK) model was built to determine anti-Xa plasma concentrations, and simulations from the model paved the way for recommending starting doses and dose increments in the product label (11, 12).

**Table 1:** Examples of response biomarkers used in pediatric drug development

Biomarker Type	Definition (49)	Application	Example
Pharmacodynamic	A response biomarker that indicates biologic activity without necessarily drawing conclusions about efficacy or disease outcomes	•Demonstrate similarity of disease pathophysiology in adults and children •Demonstrate similarity of response to therapy in adults and children •Support dose selection	•Intragastric pH for gastroesophageal reflux disease in children •Anti-Xa level for children with venous thromboembolism
Pediatric bridging biomarker	A response biomarker supported by strong mechanistic evidence and is expected to be correlated with an endpoint intended to assess clinical benefit in clinical trials, but without sufficient clinical data to show that it is a validated surrogate endpoint	•Extrapolate efficacy from adults to children for drugs that are effective in adults with similar disease	•Pulmonary vascular resistance for pediatric PAH •NT-proBNP for pediatric heart failure
Surrogate endpoint biomarker	A response biomarker that is an endpoint used in clinical trials as a substitute for a direct measure of how a patient feels, functions, or survives	•Substitute for clinical endpoint to establish efficacy in pediatric clinical trials	•Blood pressure for pediatric hypertension •Hemoglobin A1C for pediatric diabetes mellitus

*NT-proBNP* N-terminal prohormone of brain natriuretic peptide, *PAH* pulmonary arterial hypertension

Biomarkers have been used to support efficacy in a pediatric extrapolation approach. When combined with evidence of strong similarity of disease between adults and children, identification of a dose in pediatric patients that leads to a similar response in a PD biomarker could be sufficient to support efficacy. In a study of nemolizumab in adults and adolescents with atopic dermatitis, biomarkers predictive of clinical response were incorporated into a PK/PD analysis to show similarity of exposure and response between these populations (13). In another example, the similarity in exposure–response relationships of esomeprazole’s effect on intragastric pH between children and adults permitted the approval of this drug for the treatment of gastroesophageal reflux disease in children when it was not possible to evaluate the adult clinical endpoint in children (14). Importantly, for this approach to be acceptable, exploration of PD biomarkers in adult dose ranging studies should be conducted to support their potential use in a future pediatric extrapolation approach; the biomarkers should ideally be appropriate to both adult and pediatric subsets (15). Many methods can be used to explore exposure–response relationships between adult and pediatric populations, including incorporation of biomarkers into model-based approaches. A full review of potential model-based approaches is beyond the scope of this paper but are discussed in other publications (16–20).

## Justifying Reliability of Surrogate Outcome Measures and Bridging Biomarkers

An important potential use for a biomarker in overall drug development is as a replacement endpoint, provided it is established to be a validated surrogate endpoint. The use of the biomarker in this manner could meaningfully reduce the time and resources needed to conduct a pediatric trial. However, the evidentiary requirements to validate a surrogate marker for pediatric trials are as stringent as those of adult drug development programs.

### ‘A Correlate Does not a Surrogate Make’

**Fig. 1:**
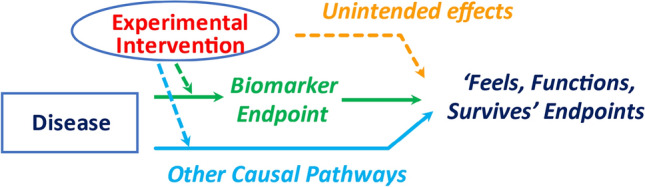
The intervention’s effect on the biomarker endpoint could overestimate or underestimate the intervention’s true clinical efficacy

Even if a biomarker at the patient-level is correlated with a measure of how a patient feels, functions, or survives (i.e., a Level III biomarker as indicated in Table 2), there are multiple reasons why the effect of an intervention on that biomarker might not reliably predict its effect on that feels, functions, or survives (FFS) measure (21–23). For example, the biomarker may not lie on the physiological pathway by which the disease process influences FFS endpoints (i.e., absence of a green solid arrow from the biomarker to the FFS endpoint in Fig. 1). Even if the biomarker does lie on the physiological pathway, the magnitude and duration of effects on that biomarker required to produce meaningful effects on the FFS endpoint are often unknown. Furthermore, the experimental intervention may have intended effects on causal pathways (Fig. 1, light blue dashed arrow) or unintended effects (Fig. 1, orange dashed arrow) that ultimately affect the FFS measure but are not captured by the biomarker.

**Table 2:** Criteria establishing levels of utility of biomarkers

Validation of the biomarker	Criteria
Level I: As a Replacement Endpoint for FFS endpoint(s) in Registrational Trial of EXP	o In depth clinical insights, including a comprehensive understanding of: ─The causal pathways of the disease process; and EXP’s intended & unintended mechanisms of action that could impact the FFS endpoint(s) o Empiric evidence, including: ─Ideally, a meta-analysis of trials establishing the net effect of EXP on the biomarker reliably predicts its net effect on the FFS endpoint(s) o Alternatively, evidence establishing the Prentice criteria: ─Strong patient-level correlation between the biomarker and the FFS endpoint(s) ─The biomarker fully captures the net effect of EXP on the FFS endpoint(s)
Level II: As a ‘*Bridging Biomarker*’ in Registrational Trials addressing Pediatric Extrapolation for the EXP	Substantive information establishing: i. The disease processes in pediatric and adult settings are closely related biologically; ii. In adults, the intervention is safe and has substantial effects on FFS measures and biomarker; iii. In adult & pediatric settings, effects on the bridging biomarker capture effects on the principal causal pathway through which the disease process meaningfully influences FFS measures; iv. In adult and pediatric settings, the EXP does not have important unintended effects on FFS measures that are not captured by the bridging biomarker; and v. By proper statistical analyses in adults, EXP’s net effect on FFS measures is consistent with what would be predicted by the level of EXP’s effect on the bridging biomarker
Level III: As being useful for understanding prognosis	Strong patient-level correlation between the biomarker and the FFS endpoint(s)

*EXP* denotes the ‘Experimental Intervention’, *FFS* denotes ‘feels, functions or survives’

Despite evidence of the experimental intervention’s impact on the biomarker and FFS endpoint in adults, as well as patient-level correlations between the biomarker and FFS measure in both adults and children, the intervention might not have favorable effects on the FFS measure in a pediatric setting, especially if the magnitude of its intended effects on causal pathways not captured by the biomarker are greater in adults than in children or its unintended effects are greater in children than in adults.

Recent experiences in the oncology setting illustrate the hazards of using biomarkers to extrapolate across classes of agents (24, 25). Biomarkers sensitive to short-term effects on tumor burden, such as objective response rate and progression-free survival, have been widely used as surrogate endpoints for effects on overall survival for chemotherapy agents, but these biomarkers frequently underrepresent the survival effects of immuno-oncology agents that have impressive effects on long-term tumor burden.

Establishing a biomarker to be a valid replacement endpoint for a direct FFS measure requires not only in depth clinical insight about causal pathways of the disease process and intended and unintended mechanisms of action of the experimental intervention, but also empiric evidence about whether the net effect of the intervention on the replacement endpoint reliably predicts its net effect on the FFS measure (i.e., a Level I biomarker as indicated in Table 2). Ideally, this would be achieved by plotting data from an array of trials to reveal that the net effect of the experimental intervention on the biomarker (plotted on the x-axis) is predictive of its net effect on the FFS endpoint (plotted on the y-axis).

Biomarkers are appropriately used as direct measures of biologic activity and are very useful as supportive measures in registrational trials or as primary endpoints in proof-of-concept trials. It is much more challenging to provide justification for their use as replacement endpoints in registrational trials, especially when evaluating new interventions in novel drug classes, and especially in pediatric settings given the scarcity of available evidence and the variable quality of data sources. On the other hand, extrapolation across populations (e.g., from adult to pediatric) through the use of a ‘bridging biomarker’ is more readily justified if the criteria provided in the next section are satisfied.

### Core Criteria Justifying Reliability of Bridging Biomarkers

The most reliable evidence about the safety and efficacy of therapeutic interventions in the pediatric setting is provided by randomized clinical trials conducted in pediatric populations, with endpoints that are FFS measures or validated surrogates. When it is not feasible to obtain such evidence, an alternative approach, if it is scientifically justified, would be to first assess efficacy and safety in adults, then establish efficacy in a pediatric population using bridging biomarkers. To justify using a bridging biomarker as a replacement endpoint for regulatory decision-making in the pediatric setting, it is proposed that the five criteria provided in Table 2 (i.e., a Level II biomarker) be satisfied. The first criterion requires substantive evidence that disease processes in pediatric and adult settings are biologically similar, and the second requires reliable evidence that the intervention is safe and effective in adults. The remaining criteria (i.e., iii, iv, and v for Level II biomarkers as indicated in Table 2) establish the biomarker to be ‘reasonably likely to predict’ meaningful effects on FFS measures (25). If these five criteria are collectively satisfied, then establishing meaningful effects on the bridging biomarker in the pediatric setting would provide sufficient evidence to extrapolate the efficacy assessment, from the adult to the pediatric setting, that the intervention has meaningful effects on the key FFS endpoints.

## Illustrating the Use of Bridging Biomarkers in Pediatric Extrapolation

We present recent case examples where efficacy established in adult populations was extrapolated to pediatric populations through the use of bridging biomarkers (Table 3).

**Table 3:** Criteria justifying the reliability of bridging biomarkers for regulatory decision-making and two examples

	Level II Validation Criteria				
Bridging biomarker and context of use	(i) The disease processes in pediatric and adult settings are biologically closely related	(ii) In adults, the EXP is safe and has substantial effects on FFS measures and biomarker	(iii) Effects on the bridging biomarker capture effects on the principal causal pathway though which the disease process meaningfully influences FFS measures	(iv) No evidence that the EXP has unintended effects on FFS that are not captured by the bridging biomarker	(v) Intervention’s effect on bridging biomarker predicts intervention’s effect on clinical endpoint
PVR obtained by right heart catheterization used for extrapolating the clinical benefit of bosentan to pediatric patients with PAH	Clinical experts agree that pediatric PAH shares common features of adult disease (27) Bosentan provides clinical benefit through pulmonary vasodilation. Similarity of response was evaluated based on change from baseline in PVR results between adults and children taking labeled doses	Six-minute walk distance was used as the primary efficacy endpoint in the adult clinical trial (28). The mean difference between the placebo and the combined bosentan groups was 44 m (95% CI 21, 67; *p* < 0.001) Treatment with bosentan led to reduction in PVR (29). The mean difference between placebo and bosentan groups was − 415 dyne*sec/cm^5^ (*p* ≤ 0.001)	PVR, by being based on pulmonary arterial pressure as well as cardiac output, is capturing effects on principal causal pathways through which the disease process impacts FFS	No unexpected deaths or serious adverse events in long-term safety follow-up in children Routine liver enzyme testing is performed in both the adult and pediatric population	Changes in PVR are correlated with changes in 6-min walk distance for individual drugs and across drug classes approved for PAH In pooled adult trials of bosentan, changes in PVR from baseline could explain 50% of the treatment effect on 6-min walk distance
NT-proBNP used for extrapolating the clinical benefit of sacubitril/valsartan to pediatric patients with heart failure	Adult heart failure with NIDCM is considered sufficiently similar to pediatric heart failure patients with NIDCM (39). Further support shown in Fig. 2 Similarity of response was evaluated based on NT-proBNP results between adults and children treated with exposure-matched doses	Time-to-first event of a composite of hospitalization for HF and CV death was the primary endpoint in the adult pivotal trial PARADIGM-HF (43). The primary endpoint occurred in 914 patients (22%) in the sacubitril/valsartan group and 1117 patients (27%) in the enalapril group (hazard ratio, 0.80; 95% CI 0.73, 0.87; *p* < 0.001) NT-proBNP was significantly lower in sacubitril/valsartan-treated patients (938 pg/ml) compared with enalapril-treated patients (1203 pg/ml); *p* < 0.001 for the difference between groups (42)	NT-proBNP is used in clinical practice to diagnose and assess disease severity. It is a strong independent prognostic factor for outcomes and is used in the management of chronic heart failure	No evidence that sacubitril/valsartan has unintended effects on measures for how the patient feels, functions, or survives that are not captured by NT-proBNP The safety profile in pediatric patients in the PANORAMA-HF interim analysis was similar to the safety profile in adults	The association of changes in NT-proBNP and hazard of hospitalization for HF or CV death was demonstrated in adults in multiple trials. In pediatric patients, changes in NT-proBNP were associated with markers of left ventricular systolic function and heart failure outcomes In PARADIGM-HF in adults, > 80% of the sacubitril/ valsartan treatment effect on the clinical endpoint was explained by change from baseline in NT-proBNP

*CI* confidence interval, *CV* cardiovascular, *EXP* experimental intervention, *FFS* denotes ‘feels, functions or survives’, *HF* heart failure, *NIDCM* nonischemic dilated cardiomyopathy, *NT-proBNP* N-terminal prohormone of brain natriuretic peptide, *PAH* pulmonary arterial hypertension, *PVR* pulmonary vascular resistance

### Bosentan in Pediatric Pulmonary Arterial Hypertension

Pulmonary arterial hypertension (PAH) is a rare progressive disorder associated with diverse cardiac, pulmonary, or systemic disease with significant morbidity and mortality. Pediatric PAH shares similar disease characteristics with adults, and clinical experts have agreed that this disease is similar enough for pediatric extrapolation (26, 27). In adults, bosentan (Tracleer®) was established to be safe and effective (28) and received regulatory approval. Its approval in children then was based on pediatric extrapolation utilizing the hemodynamic parameter, pulmonary vascular resistance (PVR) measured by right heart catheterization, as the bridging biomarker (29). The FDA review team followed a framework based on the Prentice criteria (30–32) to show that the change in PVR was very likely to predict the clinical endpoint of change in the six-minute walk distance (6MWD). The team pooled patient-level data from twelve placebo-controlled clinical trials in adults of nine approved PAH medications. Together, these data showed substantial correlation between PVR and 6MWD that was consistent across treatments, drug classes, and individual drugs. For bosentan, the team determined the proportion of the treatment effect explained by change in PVR using data from three trials in 263 adults (29). This was achieved by comparing the treatment effect estimate, i.e., placebo-corrected change in 6MWD [(36 m (95% CI 16, 55)] in a regression model using treatment group and baseline PVR as exploratory values with the treatment effect [18 m (95% CI − 0.44, 37)] estimate from the same model but with the inclusion of change from baseline in PVR as an additional covariate. In this model, 50% of the net effect of bosentan on 6MWD could be attributed to its effects on PVR. This intermediate result was further reinforced by the biological importance of pulmonary arterial pressure and cardiac output as principal causal pathways of PAH that are captured by measuring PVR, and by the absence of unexpected deaths or serious adverse events in long-term safety follow-up in children. Therefore, the review team concluded that changes in PVR would be very likely to predict the treatment effect on 6MWD. In support of this, a pediatric study of 19 patients with PAH World Health Organization functional class II/III demonstrated a statistically significant reduction in PVR from baseline at Week 12 [− 389 dyne*s/cm^5^ (95% CI − 682, − 96)], and the magnitude of change was similar to that observed in adults taking the labeled dose of bosentan (33, 34).

In 2017, the FDA-approved bosentan, the first drug for the treatment of PAH in children in the United States, based on pediatric extrapolation using PVR as the bridging biomarker (35). The use of PVR as a bridging biomarker cannot be generalized or used to test other drugs because the routine use of right heart catheterization in pediatric trials is now considered unethical due to the risk of death and serious adverse events associated with this procedure (36, 37). Therefore, pediatric extrapolation of approved PAH drugs in adults will require the development of noninvasive bridging biomarkers.

### Sacubitril/Valsartan in Pediatric Heart Failure with Dilated Cardiomyopathy

**Fig. 2:**
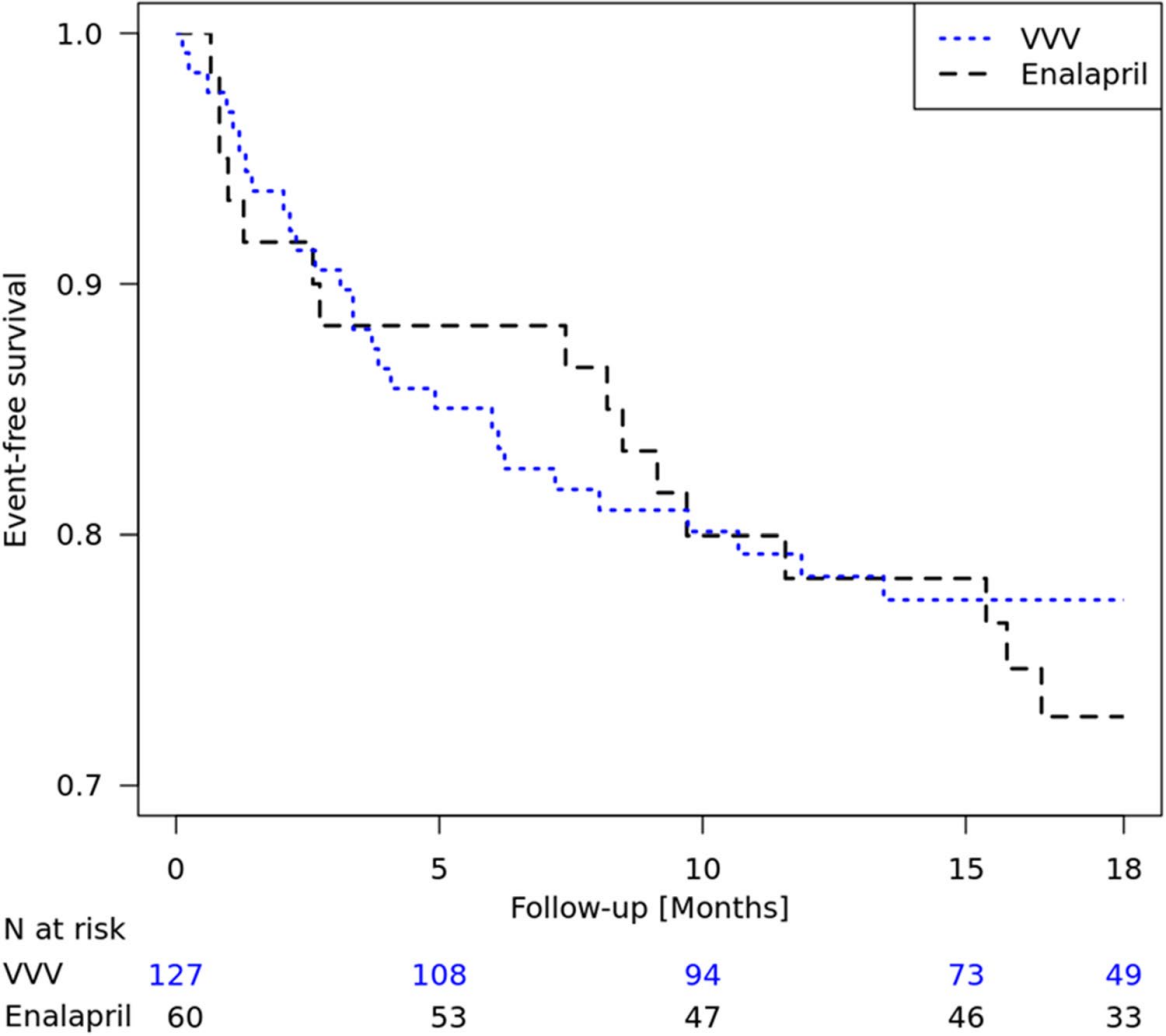
Comparison of disease progression in the PARADIGM-HF NIDCM subpopulation (adult patients ≤ 40 years old treated with enalapril) and the VVV study (pediatric patients). Disease progression: (1) PARADIGM-HF: cardiovascular death or hospitalization for heart failure; (2) VVV: hospitalization for heart failure, initiation of intravenous inotropic support, transplant listing or increasing in listing status, decompensated HF requiring mechanical circulatory support, or death. The NIH/NHLBI Pediatric Heart Network Ventricular Volume Variability Study dataset was used in preparation of this work. Data were downloaded from http://pediatricheartnetwork.org/ForResearchers/PHNPublicUseDatasets.aspx on 03/09/2019.

In 2015, sacubitril/valsartan (Entresto®) was approved in adults with chronic heart failure (HF), a disease most evident in those with reduced ejection fraction (HFrEF). At that time, pediatric HFrEF was understood to be different from adult HFrEF and therefore, a full waiver was granted. However, the manufacturer, Novartis, chose to conduct PANORAMA-HF, a pediatric trial in 360 children with systemic left ventricle HFrEF, because there was a clear unmet medical need with no intervention having regulatory approval (38). The understanding of the similarity between pediatric and adult HF changed in October 2017 with a pediatric workshop organized by the FDA (39). As a result of the workshop, adult patients with nonischemic dilated cardiomyopathy (NIDCM) were deemed sufficiently similar to patients with pediatric systemic left ventricle heart failure, a prerequisite for extrapolation. In the adult PARADIGM-HF study which was a randomized, double-blind, and prospective comparison of sacubitril/valsartan with enalapril in patients with chronic heart failure with reduced ejection fraction, more than 20% of patients had NIDCM. Sacubitril/valsartan reduced the hazard for the primary endpoint (time-to-first event of a composite of hospitalization for HF and cardiovascular death) by 25% in these patients [hazard ratio of 0.75 (95% CI 0.62, 0.91)], based on 408 primary endpoint events in 1810 patients; [data on file]. Disease similarity was further supported by an analysis comparing 127 chronic dilated cardiomyopathy pediatric patients enrolled in the Pediatric Heart Network VVV study (40, 41) to adults with NIDCM aged 40 years or younger treated with enalapril from the PARADIGM-HF study (Fig. 2). The enalapril-treated group experienced disease progression similar to that of pediatric patients in the VVV study. Multiple collaborative meetings between the FDA and the sponsor resulted in a bridging biomarker approach.

With the new understanding of disease similarity between pediatric and adult heart failure patients with dilated cardiomyopathy, Novartis explored whether a PD biomarker could be used to bridge efficacy across patient populations. N-terminal-pro brain natriuretic peptide (NT-proBNP) was considered a viable candidate to serve as a bridging marker. First, NT-proBNP is used in clinical practice to assess disease severity. It is easy to measure and is understood to be a strong independent prognostic factor for outcomes in cases of chronic HFrEF. Second, the diagnostic and prognostic value of NT-proBNP is recognized for the management of HFrEF in various international treatment guidelines. Third, the PARADIGM-HF study included a biomarker sub-study in 2080 patients that, together with data from other studies, provided sufficient evidence to support the use of NT-proBNP as a bridging biomarker. Finally, NT-proBNP was measured in the pediatric PANORAMA-HF study, including as an early readout at Week 12.

The Prentice criteria (30–32) provided a useful framework to assess whether NT-proBNP could be a viable bridging biomarker. The first two criteria were fulfilled in adults based on the PARADIGM-HF study demonstrating sacubitril/valsartan’s effect on the FFS endpoint, (time-to-first event of a composite of hospitalization for HF and cardiovascular death), and on the biomarker (corresponding to Level II, criterion ii, in Table 2) (42, 43). The third Prentice criterion, including evidence of a significant relationship between the biomarker and the FFS endpoint (Level II, criterion iii, Table 2), was covered in a PARADIGM-HF biomarker sub-study (42) and was demonstrated in other settings, both in adult (44, 45) and pediatric patients (46, 47). Finally, there was evidence that the majority of the sacubitril/valsartan treatment effect on the time-to-first event of cardiovascular death or heart failure hospitalization endpoint is explained by the change from baseline in NT-proBNP over time, addressing the fourth Prentice criterion (Level II, criteria iv and v, Table 2). For this purpose, the treatment effect estimate in the PARADIGM-HF biomarker sub-study population from a model with treatment group, region, and baseline log(NT-proBNP) as explanatory variables (hazard ratio of 0.81) was compared with the treatment effect estimate from the same model, but with the inclusion of change from baseline in log(NT-proBNP) at Month 1 as an additional covariate (Table 4). If change from baseline in NT-proBNP at Month 1 were a perfect surrogate and if these Cox regression models properly represent the causal relationships, we would expect the hazard ratio for treatment effect, when adjusted for this post-baseline variable, to be unity. In this case, due to the relationship between log(NT-proBNP) and risk of cardiovascular death or heart failure hospitalization, the hazard ratio of the residual or unexplained treatment effect in the second model was 0.96. On the log scale, 82% of the treatment effect disappeared or was explained by the effect of treatment on change from baseline to Month 1 in log(NT-proBNP). Similar assessments for subgroups defined by age and etiology yielded comparable results and supported the robustness of the finding. Based on these considerations, the FDA accepted change from baseline NT-proBNP measurement as a bridging biomarker for extrapolation of the treatment effect of sacubitril/valsartan from adults to pediatric patients. This extrapolation approach resulted in FDA approval for pediatric use. The product labeling reflects the evidence that sacubitril/valsartan reduces NT-proBNP levels and is expected to improve cardiovascular outcomes in pediatric patients with heart failure with systemic left ventricular systolic dysfunction (48).

**Table 4:** Percent of treatment effect on clinical endpoint (time-to-first event of hospitalization for HF or CV death) explained by change from baseline in NT-ProBNP at month 1—PARADIGM-HF biomarker sub-study

Explanatory variable	*N*	*n*	Model 1^a^		Model 2^a^		% treatment effect explained by ΔNT-proBNP^b^ (95% CI)
			HR (95% CI)	*p* value	HR (95% CI)	*p* value	
Sacubitril/valsartan vs. Enalapril	1007 vs. 983	197 vs. 230	0.81 (0.67, 0.98)	0.03	0.96 (0.79, 1.18)	0.72	**82.5** (1.3, 163.7)
ΔNT-proBNP					1.45 (1.29, 1.63)	< 0.0001	
Baseline log_2_(NT-proBNP)			1.49 (1.38, 1.60)	< 0.0001	1.60 (1.47, 1.73)	< 0.0001	

Cox regression of time-to-first event of hospitalization for HF or CV death

*CI* confidence interval; delta (Δ), change from baseline, *HR* hazard ratio, *log* logarithmic, *N* number of subjects, *n* number of events, *NT*-proBNP N-terminal prohormone of brain natriuretic peptide

^a^Model 1 contains treatment group, region and baseline log_2_(NT-proBNP) as explanatory variables and Model 2 contains treatment group, region, baseline log_2_(NT-proBNP) and change from baseline in log(NT-proBNP) at Month 1 (ΔNT-proBNP) as explanatory variables

^b^% reduction of treatment effect on log scale in Model 2 (after adding post-baseline change in NT-ProBNP) relative to Model 1

## Discussion

Pediatric extrapolation, including properly using insights about an intervention’s properties in adults when developing and evaluating that intervention in children, has advanced over the last 25 years. The role of biomarkers in that process has also advanced. While we have reviewed several uses of biomarkers for pediatric extrapolation, our focus has been on ‘bridging biomarkers’, i.e., biomarkers used to extrapolate insights about intervention effects from adult to pediatric settings (6).

The use of bridging biomarkers in pediatric extrapolation is distinct from other roles for biomarkers in that they are not simply a PD marker that can be used to support dose selection, and they do not necessarily meet the standards of a fully validated surrogate endpoint that can reliably predict the net effect of the intervention on FFS outcomes. It is also important to recognize that a biomarker, even when justified for use as a replacement endpoint in the clinical setting, cannot necessarily be considered a generic surrogate endpoint for a particular disease, especially when attempting to extrapolate to different agents. Interventions can differ in their intended and unintended effects, including effects on multiple causal pathways of the disease process, and the biomarker may not capture all of these effects that meaningfully influence intervention effects on FFS outcomes.

To justify the use of a bridging biomarker in registrational decision-making in the pediatric setting, the proposed biomarker should satisfy the core criteria of a Level II biomarker (Table 2). These include substantive evidence that disease processes in pediatric and adult settings are biologically similar, as well as substantial evidence that the intervention is safe and effective in adults. In addition to these important criteria, the use of a bridging biomarker should also satisfy three additional criteria in Table 2 for Level II biomarkers to increase confidence that the biomarker is likely to predict important clinically meaningful FFS effects on the disease state.

Both the bosentan and the sacubitril/valsartan examples illustrate the use of these bridging biomarker criteria in the process of pediatric extrapolation (Tables 2, 3). Data reviewed in both programs supported disease similarity between an adult and target pediatric population. Both drugs were approved for use in adults based on substantial evidence of effectiveness on FFS endpoints. For bosentan, clinically meaningful functional improvement was confirmed using 6MWD, and for sacubitril/valsartan, risks for cardiovascular death or heart failure hospitalization were reduced. Nevertheless, similarity of the disease and response to treatment between adult and pediatric patients was not sufficient to rely on PK matching alone; data from controlled trials in pediatric settings were needed to confirm efficacy in both cases. However, the endpoints used for the adult clinical trials were either not suitable for use in younger pediatric patients (6MWD for bosentan) or could not be collected in a reasonable timeframe from a small pediatric population (time to cardiovascular death or heart failure hospitalization for sacubitril/valsartan).

Therefore, the clinical development programs for these drugs sought to identify a potential bridging biomarker. For bosentan, changes in PVR are clearly part of the causal pathway of PAH, and review of available data on PVR included pooled subject-level data from clinical trials that established the relationship of PVR to the clinically meaningful endpoint of 6MWD (i.e., approximately 50% of the treatment effect is explained by changes in PVR). The available evidence provided confidence that the effects of bosentan measured by 6MWD are largely predicted by its effects on PVR.

For sacubitril/valsartan for the treatment of HFrEF, NT-proBNP was determined likely to lie in the causal pathway of HFrEF based in part on evidence showing its strong link to HFrEF clinical outcomes. Data from large studies in both adult and pediatric patients with HFrEF established a relationship of NT-proBNP with the clinically meaningful endpoint of time to cardiovascular death or heart failure hospitalization. Furthermore, due to this relationship, the analysis of data from these studies indicated that 82% of the drug’s treatment effect on the risk of cardiovascular death or heart failure hospitalization is explained by its effects on NT-proBNP. Similar to the bosentan example, these data provided confidence that the effects of sacubitril/valsartan on time to cardiovascular death or heart failure hospitalization are largely predicted by its effects on NT-proBNP.

Both examples described above benefited from trial designs that enabled the rigorous collection of evidence during the adult development program to support the use of a bridging biomarker for pediatric extrapolation. Further support for such use of bridging biomarkers could be provided by the timely availability of insights from observational registries of rare diseases in children. These registries could enable a better understanding of the natural history of rare pediatric diseases that often is not well characterized. These registries also should be initiated early to avoid prolonged delays in designing and conducting pediatric trials, and ideally should be designed to include the identification and collection of data on FFS measures and biomarkers. Even when those data provide only patient-level correlations rather than causal evidence, they would be supportive to justifying and implementing the use of a bridging biomarker and to designing and conducting pediatric trials.

Evidence to determine disease similarities between adult and pediatric populations, the safety and effectiveness of the intervention in adults, the biologic plausibility of the relationship between the biomarker and clinical endpoint, and the quantitative relationship between treatment effect on the biomarker and treatment effect on FFS measures are all necessary to justify the use of a bridging biomarker. Early planning during adult drug development and early discussions with regulatory authorities is always encouraged to ensure that there is timely and efficient development of therapies for children.

Clearly, the use of bridging biomarkers as part of a pediatric extrapolation approach offers an important opportunity to increase the efficiency and timeliness of development of pediatric interventions. This is particularly true when the pediatric populations are small (i.e., rare diseases) or when the clinical endpoints used in the adult development program cannot be used in children. Increasing the timeliness of the evaluation of pediatric interventions is particularly important for serious pediatric diseases where there are few or no approved interventions. Although the use of bridging biomarkers can reduce the time required to complete a pediatric development program, the acceptability of using a bridging biomarker must rely on evidence that pediatric extrapolation is justified and that the data to support the use of the bridging biomarker are sound and of high quality. Feasibility issues alone are not sufficient to support a bridging biomarker approach. If the bridging biomarker is not rigorously evaluated and the justification for use of pediatric extrapolation is not adequate, there is a clear risk of approving a therapy that is not effective in children. Additionally, even when a bridging biomarker approach is acceptable, other important pediatric drug development issues (e.g., dose selection; formulation development; safety data collection) must also be addressed.

In summary, we provide a set of core criteria to justify the use of a bridging biomarker as part of a pediatric extrapolation approach along with recent examples that illustrate this method. Importantly, this framework relies on foundational criteria, including a similarity of disease and response to intervention between an adult and pediatric population to support pediatric extrapolation and substantial evidence of effectiveness demonstrated in adults. Additional data needed to meet the core criteria should ideally be considered for collection in pediatric rare disease registries and during adult drug development to avoid delays in the design and conduct of pediatric trials. We recognize that therapies first approved in adults are then used off-label in children because practitioners and guardians who are making decisions about their child’s health care want to have a choice. Yet, children and their families deserve the opportunity to make informed decisions about their health care based on reliable data. Ultimately, the use of bridging biomarkers is intended to increase the timeliness of adequately evaluating therapies for use in children so that practitioners, pediatric patients, and their families can make informed choices about their health care based on the availability of reliably evaluated and approved therapies.
